# Supplementary material for: Building Consensus on the Point-of-Care Ultrasound Skills Required for Effective Healthcare Service Delivery at District Hospitals in South Africa: A Delphi Study
Source: Int J Environ Res Public Health. 2023 Nov 30;20(23):7126. doi: 10.3390/ijerph20237126 (PMC10705875; doi:10.3390/ijerph20237126)
Supplement: Supplementary file 1 [file ijerph-20-07126-s001.zip › Supplementary material 2 - expert consensus on all skillsets.pdf]

Supplementary material 2: Expert consensus on POCUS skillsets

1.1.1	Identification of the presence of an intrauterine pregnancy	Essential	R1
1.1.2	Determine the viability of an intrauterine pregnancy	Essential	R1
1.1.3	Detection of the foetal heart rate using M-Mode	Essential	R2
1.1.4	First trimester pregnancy gestational age assessment by crown rump length detection	Essential	R1
1.1.5	Recognition of molar pregnancy	Essential	R1
1.2.1	Determine placental position	Essential	R1
1.2.2	Determine the foetal presentation	Essential	R1
1.2.3	Perform a gestational age assessment and foetal weight estimation using abdominal circumference (AC), biparietal diameter (BPD), and femoral length (FL).	Essential	R1
1.2.10	Confirmation of foetal death	Essential	R1
1.2.4	Assess the amniotic fluid volume using either the four-quadrant calculation or deepest single pocket approach	Essential	R2
1.2.5	Assess the placenta for features of placental abruption	Essential	R2
1.2.8	Assess foetal well-being using the biophysical profile	Essential	R4
1.2.9	Assess foetal well-being during third trimester using umbilical artery doppler	Essential	R5
1.3.1	Confirmation of intrauterine device (IUD) position	Essential	R3
1.3.2	Measurement of endometrial thickness	Essential	R2
1.3.3	Assessment of an adnexal mass: simple, complex and haemorrhagic cysts	Essential	R5
2.1	Detection of a pericardial effusion	Essential	R1
2.10	Measurement of inferior vena cava (IVC) diameter and collapsibility to approximate volume status	Essential	R5
2.2	Assessment of global left ventricle contractility (hyperdynamic / normal / decreased)	Essential	R4
3.1	Assessment of free fluid in the abdominal cavity	Essential	R1
3.2	Assessment of free fluid around the heart with a sub-xyphoid view	Essential	R1
3.3	Assessment of a pneumothorax	Essential	R2
3.4	Assessment of a haemothorax	Essential	R2

4.1.1	Detection of an abdominal aortic aneurysm	Essential	R3
4.2.1	Assess the gallbladder for cholelithiasis	Essential	R2
4.2.2	Assess for acute cholecystitis	Essential	R2
4.2.3	Assess for common bile duct (CBD) obstruction (Choledocholithiasis)	Essential	R5
4.2.4	Assess for hepato-splenomegaly	Essential	R5
4.4.1	Assess renal size	Essential	R4
4.4.5	Identify a testicular torsion	Essential	R2
5.1	Identify lower extremity DVT in low-risk cases with 2-Zone discrimination technique	Essential	R1
5.2	Identify lower extremity DVT with colour Doppler, and graded compression of the entire limb	Essential	R3
7.1	Identification of pulmonary oedema	Essential	R5
7.3	Identification of pneumothorax	Essential	R2
7.4	Identification of pleural effusion or haemothorax	Essential	R2
9.1	Thoracentesis	Essential	R2
9.2	Paracentesis	Essential	R2
9.3	Peripheral IV placement	Essential	R2
9.4	Central line placement	Essential	R1
9.7	Foreign body identification and removal	Essential	R2
9.8	Fine needle aspiration/biopsy	Essential	R2
10.1	FAST/E-FAST: Focused assessment with sonography for trauma	Essential	R1
10.2	RUSH: Rapid ultrasound for shock and hypotension	Essential	R2
10.3	BLUE: Bedside lung ultrasound in emergency	Essential	R2
10.4	CLUE: Cardiac limited ultrasound exam	Essential	R2
4.4.2	Identify and grade hydronephrosis	No agreement	R3
4.4.3	Identify urinary retention and post-void residual volume	No agreement	R4
6.7	Identification of rotator cuff injury	No agreement	R3
1.2.7	Determine foetal gender after 18 weeks	Non Essential	R3

2.9	Assessment of right ventricle function using TAPSE	Non Essential	R4
6.10	Assessment of carpal tunnel syndrome	Non Essential	R4
6.6	Identification of tendonitis/tendinopathy	Non Essential	R4
6.8	Identification of a ligament injury of the knee	Non Essential	R4
6.9	Identification of a ligament injury of the ankle	Non Essential	R4
7.6	Identification of interstitial pneumonia	Non Essential	R3
7.8	Identification of chronic interstitial lung disease	Non Essential	R4
8.1	Identification of detached retina	Non Essential	R3
8.2	Identification of vitreous detachment	Non Essential	R3
8.3	Identification of vitreous haemorrhage	Non Essential	R3
8.4	Identification of intraocular foreign body	Non Essential	R3
8.5	Measurement of posterior ocular nerve sheath diameter for assessment of intracranial pressure (papilledema)	Non Essential	R4
8.6	Identification of a lens dislocation	Non Essential	R2
9.5	Lumbar puncture	Non Essential	R3
1.2.6	Evaluation of cervical length to assess cervical insufficiency	Optional	R3
1.3.4	Identification of an ovarian torsion	Optional	R4
1.3.5	Assessment of a breast mass	Optional	R4
10.5	FEEDS criteria: To determine presence IUP	Optional	R4
2.3	Calculation of left ventricle ejection fraction through radial contractility (Teicholtz)/ mitral annular plane systolic excursion (Mapse)	Optional	R4
2.4	Assessment of left ventricular hypertrophy	Optional	R3
2.5	Assessment of diastolic dysfunction	Optional	R4

2.6	Assessment of regional wall motion abnormalities	Optional	R3
2.7	Assessment of valvular abnormalities	Optional	R3
2.8	Assessment of right ventricle size and strain and the possibility of PE in the appropriate clinical setting	Optional	R5
4.1.2	Detection of an abdominal aortic dissection	Optional	R3
4.1.3	Detection of a proximal aortic root aneurysm or dissection	Optional	R4
4.3.1	Assess for appendicitis	Optional	R3
4.3.2	Identify an abdominal wall hernia	Optional	R4
4.3.3	Identify an inguinal hernia	Optional	R4
4.4.4	Identify a varicocele	Optional	R4
4.4.6	Identify epididymo-orchitis	Optional	R3
4.4.7	Assess prostate volume	Optional	R4
5.3	Identify upper extremity DVT with colour Doppler, and graded compression of the entire limb	Optional	R3
6.1	Differentiating between cellulitis and an abscess in soft tissue	Optional	R4
6.2	Identification of a foreign body	Optional	R4
6.3	Identification of a joint effusions/bursitis	Optional	R3
6.4	Identification of long bone fractures	Optional	R5
6.5	Identification of tendon rupture/tear	Optional	R3
7.2	Identification of a lobar pneumonia	Optional	R4
7.5	Assessment of pleural mass	Optional	R4
7.7	Identification of Acute Respiratory Distress Syndrome (ARDS)	Optional	R4
9.10	Peripheral nerve blocks	Optional	R3
9.6	Knee aspiration and injection	Optional	R4
9.9	Shoulder, ankle, hip, wrist aspiration, and injection	Optional	R2
